# Novel electrotactile brain-computer interface with somatosensory event-related potential based control

**DOI:** 10.3389/fnhum.2023.1096814

**Published:** 2023-03-23

**Authors:** Andrej M. Savić, Marija Novičić, Olivera Ðorđević, Ljubica Konstantinović, Vera Miler-Jerković

**Affiliations:** ^1^School of Electrical Engineering, University of Belgrade, Belgrade, Serbia; ^2^Faculty of Medicine, University of Belgrade, Belgrade, Serbia; ^3^Clinic for Rehabilitation “Dr. Miroslav Zotović”, Belgrade, Serbia; ^4^Innovation Center of the School of Electrical Engineering, University of Belgrade, Belgrade, Serbia

**Keywords:** brain computer interface (BCI), event-related potentials (ERP), electrical stimulation, somatosensory evoked potential (SEP), tactile attention, tactile BCI, machine learning

## Abstract

**Objective:**

A brain computer interface (BCI) allows users to control external devices using non-invasive brain recordings, such as electroencephalography (EEG). We developed and tested a novel electrotactile BCI prototype based on somatosensory event-related potentials (sERP) as control signals, paired with a tactile attention task as a control paradigm.

**Approach:**

A novel electrotactile BCI comprises commercial EEG device, an electrical stimulator and custom software for EEG recordings, electrical stimulation control, synchronization between devices, signal processing, feature extraction, selection, and classification. We tested a novel BCI control paradigm based on tactile attention on a sensation at a target stimulation location on the forearm. Tactile stimuli were electrical pulses delivered at two proximal locations on the user’s forearm for stimulating branches of radial and median nerves, with equal probability of the target and distractor stimuli occurrence, unlike in any other ERP-based BCI design. We proposed a compact electrical stimulation electrodes configuration for delivering electrotactile stimuli (target and distractor) using 2 stimulation channels and 3 stimulation electrodes. We tested the feasibility of a single EEG channel BCI control, to determine pseudo-online BCI performance, in ten healthy subjects. For optimizing the BCI performance we compared the results for two classifiers, sERP averaging approaches, and novel dedicated feature extraction/selection methods *via* cross-validation procedures.

**Main results:**

We achieved a single EEG channel BCI classification accuracy in the range of 75.1 to 88.1% for all subjects. We have established an optimal combination of: single trial averaging to obtain sERP, feature extraction/selection methods and classification approach.

**Significance:**

The obtained results demonstrate that a novel electrotactile BCI paradigm with equal probability of attended (target) and unattended (distractor) stimuli and proximal stimulation sites is feasible. This method may be used to drive restorative BCIs for sensory retraining in stroke or brain injury, or assistive BCIs for communication in severely disabled users.

## 1. Introduction

Brain-computer interfaces (BCIs) enable a direct control of external device by recognizing changes in user’s brain activity, and electroencephalography (EEG) as a non-invasive technique for recording electrical brain activity still remains most frequently used measurement modality in designing BCI systems (Abiri et al., 2019). Various EEG-based paradigms and signals have been attempted in BCI control such as event-related potentials (ERP), oscillatory brain activity, steady-state evoked potentials (SSEP), and slow cortical potentials (Ramadan and Vasilakos, 2017). BCI systems with control based on ERP or SSEP are classified as “reactive BCIs,” because brain activity is modulated in reaction to an external stimulus generated by the BCI system (Steinert et al., 2019). More specifically, the user performs selective attention task, creating a mismatch in event-related/evoked brain responses associated with attended vs. unattended stimuli (Sellers et al., 2012). ERP-based BCI control relies on various recording paradigms and related signal-components such as: P300, N170, N200, motion visual evoked potential, miniature asymmetric visual evoked potential, and error-related potential (Xiao et al., 2020).

Depending on the nature of the evoking stimulus, reactive BCIs can be visual, auditory, and tactile. Tactile BCIs are still the least studied among all reactive BCIs probably due to a need for a dedicated stimulation device which requires more complex hardware than using a simple computer screen, light- or a sound-source (Brouwer and van Erp, 2010). Tactile BCIs utilize somatosensory stimuli delivered by vibration devices or electrical stimulation. Somatosensory stimulation can elicit different responses measured by EEG, among which are somatosensory evoked potentials (SEP). Routinely used SEPs in clinical practice are elicited by electrical stimulation to the peripheral nerves and consist of a series of waves that reflect sequential activation of neural structures along the somatosensory pathways (Toleikis, 2005). However, tactile BCIs rely on a somatosensory evoked electrophysiological responses recorded at cortical level. The stimulation rate can be set to evoke either transient or periodic brain responses. Transient stimuli induce cortical SEP waveforms comprised of series of signal components (signal deflections or inflexions) usually analyzed in in time domain (Petit et al., 2021). With a continuous stimulation and shortening of interstimulus interval, the somatosensory system cannot go back to an idle state (Namerow et al., 1974) and such signals are referred to as steady-state somatosensory-evoked potentials (SSSEP), and are usually analyzed in frequency domain (Tobimatsu et al., 1999). SSSEP-based control has been previously explored in various studies (Petit et al., 2021). Usually reported drawbacks of SSSEP BCIs are lower accuracy for tactile SSEP modality, lower bit-rates and the need for positioning of the stimulation points on distant body parts (exp. left vs. right hand) in order to increase classification accuracy.

Contrary, when stimulation rate is set to evoke transient SEP stimuli at cortical level, and such stimulation is coupled with cognitive task, the resulting responses can be termed somatosensory event-related potentials (sERP), even though the methodology of their induction and recording may be identical to scalp measurements of classical SEPs (Josiassen et al., 1990). Tactile BCI control using transient stimuli that induce sERP (such as somatosensory P300) have previously been attempted, but in a very limited number of studies. Brouwer and van Erp (2010) used vibrotactile stimuli with 7 tactors positioned around the subjects’ trunk. In this study the target stimuli were delivered to only one out of 7 tactors which creates a P300 response due to lower probability of target stimuli. Kaufmann et al. (2014) proposed BCI wheelchair control based on tactually-evoked ERPs. They employed vibrotactile stimulation at 4 locations: left thigh, right thigh, abdomen and lower neck, while the target tactor was one of the 4 positions. Guger et al. (2017) employed vibrotactile stimulation at left/right wrists in healthy controls and ALS patients to elicit P300 responses, while Spataro et al. (2018) used the same approach to assess somatosensory discrimination in unresponsive wakefulness syndrome. Chu et al. (2021) proposed tactile oddball paradigm employing vibrotactile and electrical stimuli, at left- and right-hand fingers with lower probability of the target stimulus (one target in six stimuli) and used oscillatory EEG (event-related spectral perturbations) for classification.

Therefore, tactile BCI control using sERP was explored in much smaller scope and less systematically compared to SSSEP BCI work, but this type of control using transient stimuli may introduce multiple benefits over the continuous stimulation and SSSEP control. Some drawbacks of SSSEP BCIs are the need for screening for subject specific resonant frequencies of the steady-state responses (Breitwieser et al., 2012), the difficulty of the tactile attention task with parallel streams of continuous stimuli, BCI illiteracy related generally to steady-state responses, lower classification accuracies for SSSEP, while higher accuracies are achieved only for stimulation of distant body regions (right vs. left hand), (Ahn et al., 2016; Petit et al., 2021).

ERP-based control can be employed for overcoming some of these drawbacks. The first benefit of ERP-based control is the possibility of using more proximate stimulation hotspots since the stimulation pulses are delivered in sequential manner to each location, so counting the number of stimuli per location is possible as mental strategy for attentional focus toward a target hotspot. Lower performance in some subjects can be addressed with averaging techniques for increasing the sERP signal to noise ratio. Moreover, SEPs are known to correlate with sensory cortex excitability (Höffken et al., 2007) and have been previously used for its assessment. Therefore, sERP-based BCI provide an opportunity for designing a neurofeedback paradigm to modulate sERP components solely with mental attention strategies, which may have a direct impact in the fields sensory training, plasticity and neurorehabilitation.

This study aimed to explore the feasibility of a novel BCI prototype based on sERP in healthy users. Here we introduce and describe a dedicated hardware setup with electrotactile stimuli and tests of different feature extraction/selection methods and classification approaches for optimizing novel sERP-based BCI performance.

The main novelties introduced within our BCI prototype are:

• compact stimulation hardware setup employing 2 stimulation channels formed by 3 stimulation electrodes placed at the proximal locations over the user’s forearm muscles;
• novel BCI control signal, somatosensory ERP, elicited by stimulating the mixed nerves of the forearm (instead of sensory nerve branches used for classical SEP recordings);
• novel tactile BCI paradigm employing equal probability of target and distractor stimuli instead of a classical oddball paradigm with rare and frequent stimulus;
• dedicated feature extraction and selection methods for sERP-based control.

Our main hypothesis was that a mismatch in sERP when attending the stimuli delivered at one of the two spatially proximal locations on the user’s forearm, with equal probability of stimuli occurrence, can result in single EEG channel BCI with accuracy over 75% in all subjects.

## 2. Materials and methods

### 2.1. Subjects

Ten healthy right-handed subjects (9 male and 1 female, aged between 22 and 32) participated in this study. Participants volunteered for the tests, and were recruited among the students and employees of the University of Belgrade. Participants were without a history of neurological disorders or somatosensory deficits, and with normal or corrected to normal vision. Subjects had no previous experience with EEG measurements or BCI experiments. This study is in accordance with ethical guidelines as defined by the Declaration of Helsinki. Study was approved by local ethical committee (no. 03-1514/1). All participants gave written informed consent.

### 2.2. Instrumentation and experimental setup

The EEG signals were recorded with the combination of g.USBamp electrophysiological signal amplifier and active electrodes with preamplification (g.GAMMAcap2, g.tec GmbH, Austria). Signals were acquired from six EEG recording sites positioned according to the 10–20 system: C3, Cz, C4, CP3, Pz, and Fp1. The reference electrode was placed on the left earlobe and the ground location was AFz. Signal from Fp1 location was used to register ocular artifacts. This subset of EEG channels have been selected based on the review of topography of sERP responses and the main sources of EEG activity which are expected in the contralateral somatosensory cortex (Josiassen et al., 1990; Li et al., 2019; Grigoryan et al., 2020; Chu et al., 2021). The signals were digitized with a 1,200 Hz sampling rate and the amplifier was configured to use embedded notch filter at 50 Hz.

Electrical stimulation (ES) device used was an eight-channel electrical stimulator, MOTIMOVE (3F–Fit Fabricando Faber, Serbia), while 2 channels were employed for stimuli delivery in our study. Three electrodes for ES were positioned on the right forearm of the subjects in order to obtain 2 stimulation channels. Two active (stimulating) electrodes of 1 cm diameter were used on the dorsal and volar surface the right forearm. A single common indifferent electrode of 2.5 cm diameter for both ES channels was located at volar aspect of the right wrist. All ES electrodes were of round shape (Axelgaard Manufacturing Co., Ltd.). Stimuli used in this study were single, constant current, compensated biphasic pulses with exponential discharge. Current pulse duration was 0.25 ms in the active phase while the inter-pulse interval was set to 700 ms.

### 2.3. Experimental protocol

The participants were comfortably seated in a chair with a computer screen in front of them at approximate 1 m distance. At the start of the experiment, all EEG and ES electrodes were positioned, quality of EEG signals was checked, and individual stimulation amplitudes for both ES channels were set. Experimenter operated the BCI system (EEG amplifier and ES) *via* custom graphical user interface developed for this purpose in MATLAB R2020a (MathWorks Inc., Natick, MA, USA). The software included EEG acquisition, stimulation control and synchronization between the events. Subjects were instructed to limit the body movements (arms, legs, face, and eyes), and to avoid excessive and systematic blinking during EEG recordings. Fixation cross displayed on the screen was introduced to limit the ocular movements.

**FIGURE 1:**
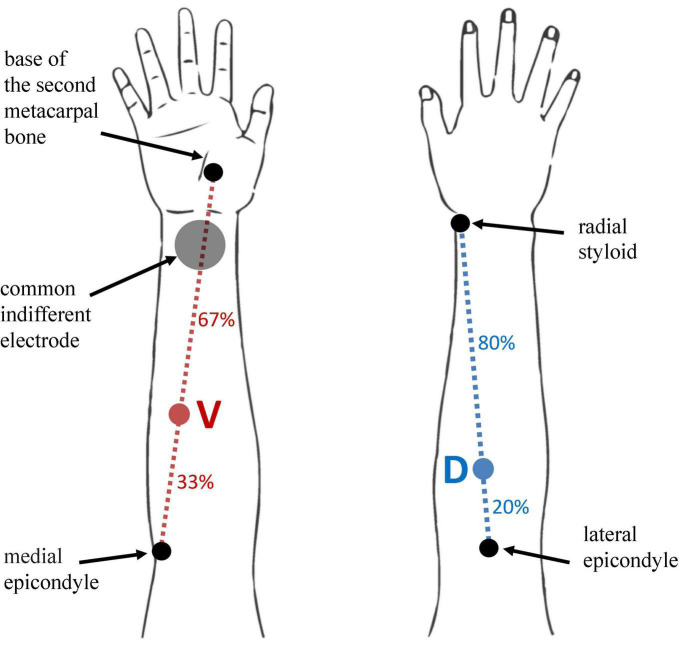
Schematics of D and V location positions on the user’s forearm, and indifferent electrode. Location D is identified at proximal 20% of the line length from the lateral humeral epicondyle to the radial styloid (blue dotted line) with the forearm in pronation. Location V is identified at proximal 33% of the line connecting the medial epicondyle and the base of the second metacarpal bone with the forearm in supination. Locations D and V are marked with blue and red colored circles on the dotted lines of the same color, respectively. The anatomical markers for determining the reference lines are marked with black circles and labeled accordingly. The indifferent electrode position over the volar aspect of the wrist is marked with gray full circle.

Two active (stimulating) electrodes were placed on the dorsal and volar surface of the right forearm. A single common indifferent electrode for both ES channels was placed on the volar aspect of the right wrist. Dorsal active ES electrode was placed over the extensor carpi radialis muscle (location D) while the volar active ES electrode was placed over the flexor carpi radialis longus (location V). Location D was first identified at proximal 20% of the line length from the lateral humeral epicondyle to the radial styloid with the forearm in pronation (Liu et al., 1997). Location V was first identified at proximal 33% of the line connecting the medial epicondyle and the base of the second metacarpal bone with the forearm in supination (Song et al., 2014). Positions were confirmed by palpation during wrist extension and flexion. Determination of locations D and V and electrode placement was conducted by an experienced clinician and researcher, specialized in physical medicine and rehabilitation. Schematics of electrode site locations for both D and V locations is presented in Figure 1.

Motor threshold for was obtained for each ES channel by increasing the pulse amplitude starting from 5 mA, in 1 mA steps. Stimulation was increased over the motor threshold to inspect the muscle activation associated with each stimulation location (D and V). If the stimulation didn’t selectively activate the target muscles, stimulation electrodes were manually repositioned until selective activation of flexor carpi radialis longus and extensor carpi radialis inspected by an experienced clinician was successfully obtained. When the expected muscle twitch responses were observed at each location, the stimulation amplitude was reduced by 1 mA in order to avoid any visible motor response while preserving the pronounced sensation. The next step was balancing the sensation intensities over two channels by decreasing the stimulation amplitude at the channel which induced the stronger sensory response (reported by the subject) in order to ensure the most similar subjective feeling of electrical stimulation intensity at both sites. The final stimulation intensities for both sites were noted by the experimenter.

**FIGURE 2:**
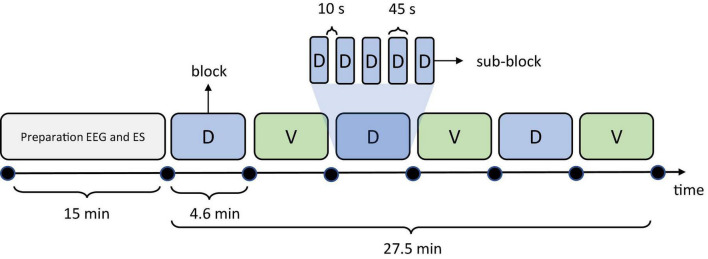
Timeline of the test depicting the durations of experimental phases (blocks). Colored shapes (light blue and light green) represent the 6 experimental blocks in which the subjects attended the stimuli delivered at D and V locations, respectively. One example of division of a single D block into 5 sub-blocks is presented also.

The experiment comprised six blocks. In each block, 300 stimuli were delivered in pseudo randomized order to locations D and V. Randomization was performed in such manner that prevented more than 3 consecutive stimuli delivery at the same location. The subjects were instructed to perform a tactile attention task by attending the stimuli delivered to only one location (targets) while attempting to ignore the stimuli delivered to the other location (distractors). We instructed the subject to silently count the number of stimuli delivered to target location. The target/distractor locations switched between the blocks, so blocks 1, 3, 5 and blocks 2, 4, 6 were associated with different target locations, while the staring target location was randomized over subjects. In order to facilitate the counting task and avoid high stimuli counts per block we have divided each block in which a single stimulation site was attended to 5 sub-blocks of 25–35 stimuli (60 in total) pseudo-randomized per stimulation site followed by 10-s pause in which subject reported the counted number of attended stimuli. In this manner we aimed to ensure that the subjects were attending the correct target location and that the counting task was successful. Also, by randomizing the number of stimuli delivered at both locations within a sub-block, we aimed to decrease the habituation effect which may arise from subject’s expectation of the same number of stimuli per sub-block. However, the probability of stimuli over all blocks was balanced. Timeline of the test is shown in Figure 2. The total number of stimuli in one subject was 1,800, with 900 stimuli delivered per location (ES channel). During electrotactile stimulation, the subject’s gaze was directed toward a fixation cross in the middle of the monitor. During the 10-s pauses between the sub-blocks the message “say number of stimuli” appeared on the screen instructing the subjects to report the counted number of stimuli followed by a countdown from 3 to 0 which indicated the start of the next block.

### 2.4. Data preprocessing

EEG was processed offline in order to obtain somatosensory event-related potential (sERP) responses elicited by electrical stimuli at each location. For easier explanation, individual responses to each sensory stimulus (or single response trials) are termed SEP, and averaged single-trial SEPs are termed sERP.

Recorded EEG data from all channels was first bandpass filtered using a 2nd order Butterworth filter with cut-off frequencies of 0.1 and 25 Hz and segmented to 500 ms epochs with 100 ms pre-stimulus baseline and 400 ms post-stimulus window. All epochs were baseline corrected by subtracting from the 400 ms window the mean value of the 100 ms baseline for each channel. Epochs containing ocular and other high amplitude artifacts were rejected by applying the threshold of 50 μV on an absolute value on all EEG channels and threshold of 80 μV on Fp1 channel for identifying blink artifacts. If an absolute value on either of the EEG epochs crossed the EEG amplitude threshold or the absolute value of the Fp1 channel crossed the EOG threshold, the epoch was rejected from further analysis.

Remaining epochs were divided into four SEP waveform clusters derived from 2 stimulation locations (D or V location) and 2 experimental conditions (attended D or attended V stimuli). Consequently, four SEP waveform clusters were extracted: attended D location with stimulus delivered to D (ADSD); attended D, stimulated V (ADSV); attended V, stimulated V (AVSV); and attended V, stimulated D (AVSD). In each subject, each SEP cluster contained more than 400 individual SEP waveforms after the artifact rejection (450 before the rejection).

In order to extract relevant features for classification of users’ tactile attention toward one of the 2 stimulated locations we employed averaging of single-trial SEPs in order to increase signal-noise ratio. Since the speed of the BCI operation directly depends on the number of trials to be averaged we have tested 3 options, average of 3, 5, and 10 single-trial SEPs per location (SEP3, SEP5, and SEP10, respectively).

The averages were formed from consecutive preprocessed, noise-free single-trial SEPs of each of the four waveform clusters for a single subject in order to form average sERPs for each cluster. Since after trial rejection the number of remaining single-trials may have been unbalanced, we rejected the last trials of larger clusters in order to obtain an equal number of average SEPs per cluster. Each averaged sERP waveform included 400 ms post-stimulus interval (480 samples), and those were downsampled by factor 8, resulting in 60 samples for the post-stimulus interval (equivalent to a new sampling frequency of 150 Hz), used in the next steps in forming the feature vectors for classification.

Difference sERP waveforms for each experimental condition [diffsERP(AD) and diffsERP(AV)] were formed by subtracting sERP obtained for location V from sERP obtained for location D, within the same condition (attended location) described by the following expressions:


diffsERP⁢(AD)=sERP⁢(ADSD)-sERP⁢(ADSV)

diffsERP⁢(AV)=sERP⁢(AVSD)-sERP⁢(AVSV)

Since a novel sERP paradigm was tested in this study, it was important to rigorously control the data quality for any sources of noise. Three experts have been involved in validating the recorded EEG and processed SEP/sERP data. One expert in EEG signal processing, SEP and ERP has processed and validated the dataset, confirming the obtained sERP waveforms are indeed of neurophysiological origin. In order to decrease the risk of bias, two independent ERP experts not involved in this study were asked to assess the EEG data quality, single trial SEPs and averaged sERP waveforms (of all conditions) for each subject/channel.

### 2.5. Feature extraction and selection approaches

In this study we explored 2 feature extraction approaches and one feature selection approach on average sERPs from 4 waveform clusters. Feature extraction/selection was performed on each EEG channel separately, since in this study we aimed to explore the feasibility of a single EEG channel electrotactile BCI control. Therefore, combination of features from different channels was out of the scope of this study. The feature extraction and selection methods applied in this study are novel and dedicated for particular BCI setup and sERP control scenario.

In the first feature extraction approach (F1), 2 feature vectors were formed by creating a single feature array of sERP amplitude values (for a single channel) by joining the consecutive pairs of averaged and downsampled sERP waveforms from clusters ADSD, ADSV for class AD; and AVSD, AVSV for class AV. Consequently, each feature vector consisted of 120 samples where the first 60 samples were sERP responses elicited by stimulation of D location while the second 60 were averaged sERP responses elicited by stimulation of V location.

In the second feature extraction approach (F2), feature vectors were formed from 2 difference sERP waveforms, associated with two experimental conditions/classes, i.e., diffsERP(AD) and diffsERP(AV). Each feature vector for this approach consisted of 60 samples, which is the length of each diffsERP waveform extracted from averaged downsampled sERPs.

Finally, we tested a fine-tuning method of feature selection for each EEG channel by employing an iterative approach. Feature selection was performed on both feature extraction methods (F1 and F2) resulting in reduced number of final features for each method (FS1 and FS2, respectively). Firstly, for each EEG channel we calculated the difference-wave between 2 conditions [diffsERP(ADAV)] as follows:


diffsERP⁢(ADAV)=diffsERP⁢(AD)-diffsERP⁢(AV)

To select relevant sERP features for further classification we employed an amplitude threshold in μV starting from 0, with an increment of 0.1 applied on the rectified diffsERP(ADAV) signal. For each threshold value, the indexes of signal samples which are equal or exceed the current threshold value are identified. We formed a feature vector of selected features for current channel and iteration (threshold value) in the same manner as previously explained. In case of FS1 the feature vector for each threshold value was formed by creating a single array of mean sERP amplitude values at selected indexes by joining (ADSD, ADSV) and (AVSD, AVSV), for classes AD and AV, respectively. In case of FS2 the feature vector for each threshold value was formed by extracting the sERP amplitudes of diffsERP(AD) and diffsERP(AV), for classes AD and AV, respectively.

### 2.6. Classification approaches

In this study we aimed to compare different classification approaches in order to explore the possibility of novel online BCI control using electrotactile sERP mismatches induced with tactile attention task. Two most popularly used classifiers in BCI applications have been tested: support vector machine (SVM) and linear discriminant analysis (LDA) (Lotte et al., 2018). The SVM classifier was implemented with a Gaussian kernel. Kernel scale parameter was optimized using a heuristic procedure, while the median of the Euclidean distance between a subsample of the data was used to update the kernel scale. The Box-constraint was set to default value. The LDA classifier was implement with solver singular value decomposition, as recommended for data with a large number of features. Each classifier performance was tested using leave-one-out cross-validation procedure while adopted performance measure was classification accuracy.

It is important to note that feature extraction/selection was performed for each iteration of the leave-one-out procedure for the training set and test set separately. More specifically, calculation of diffsERP(ADAV) and feature fine-tuning was performed on the test set in each leave-one-out iteration so that the training set and test set in each iteration are independent. Therefore, the testing procedure of the classifier is fully translatable to online BCI approach.

### 2.7. Data analysis

The overall aim of the data analysis was to test the effects of the following factors on the BCI performance (tactile attention task classification accuracy):

• classifier type (LDA, SVM).
• number of trials for averaging (SEP3, SEP5, SEP10),
• A total of 4 different feature vector generation methods from 2 feature extraction approaches with and without feature selection (F1, F2, FS1, and FS2).

The two-step iterative analysis was adopted, with a final aim to identify the optimal combination of classifier, number of trials for averaging, and feature vector generation. Two-way repeated measures ANOVA were used to assess the differences in accuracy between classifiers (LDA and SVM) and number of averaged trials (SEP3, SEP5, and SEP10) with significance threshold *p* < 0.05. *Post-hoc* analysis of trial averaging effect was conducted with Wilcoxon signed-rank test by comparing SEP3, SEP5 and SEP10. Significance threshold was *p* < 0.017, with Bonferroni correction applied (0.05/3). In this step the combination of classifier and sERP averaging approach, resulting in significantly higher accuracies than the other combinations, was selected for further analysis. Four combinations of feature extraction/selection methods (F1, F2, FS1, and FS2) were compared using 1-way repeated measures ANOVA (significance threshold: *p* < 0.05). *Post-hoc* analysis of feature extraction/selection methods was conducted with Wilcoxon signed-rank test. Significance threshold was *p* < 0.0083, with Bonferroni correction applied (0.05/6).

## 3. Results

**FIGURE 3:**
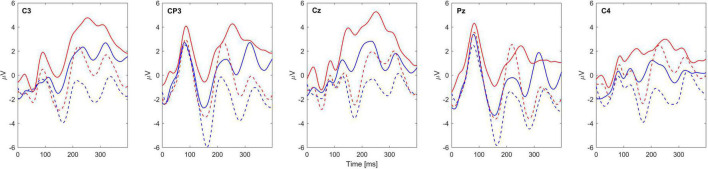
Grand-average sERP waveforms of subject 2 for 5 EEG channels. Blue lines represent the sERPs associated with stimulation of the radial nerve (D location), while the red lines represent the sERPs associated with stimulation of the median nerve (V location). The solid line represents the attended condition while the dashed line represents the unattended condition. Channel labels are given within each subplot. Zero marks the stimulus onset.

Figure 3 shows the grand-average sERPs over all blocks of one subject tested (ID: 2) for locations D and V and the AD and AV conditions.

Detailed analysis of obtained sERP morphology and topography was out of the scope of this study, and displaying the waveforms in Figure 3 is for exemplary purpose. However, it is important to note that the data of all subjects follow the similar pattern, i.e., overall increase of absolute signal amplitude for attended compared to unattended condition in both stimulation sites. Also, presence of typical ERP components such as P1, N1, P3, and their latency alignment between conditions is also visible on Figure 3, further validating that the obtained responses are indeed of neurophysiological nature.

**FIGURE 4:**
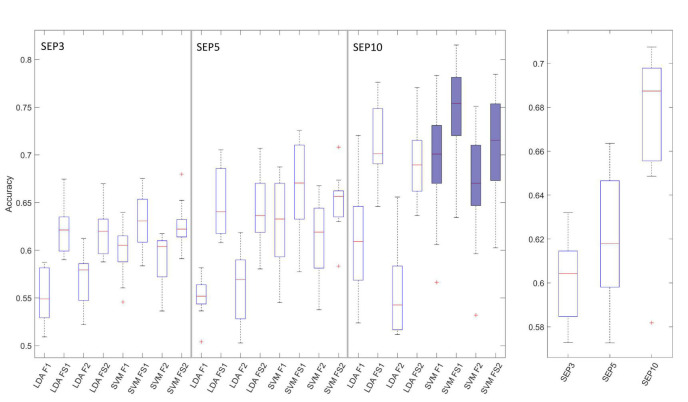
Boxplots of the BCI performance (accuracy) results over all channels. The left graph shows results for all tested combinations of classifiers (LDA and SVM) and feature extraction/selection methods (F1, F2, FS1, and FS2). Left graph is divided into 3 groups of boxplots (SEP3, SEP5, and SEP10 results), from left to right, respectively. Combination of SVM, SEP10 and 4 feature extraction methods selected for further analysis (last 4 boxplots) are filled with blue color. The right graph shows grouped result over 3 trial-averaging options (SEP3, SEP5, and SEP10). The horizontal line denotes median, boxes are interquartile ranges, whiskers show minimum and maximum values, and outliers are marked with red crosses.

The BCI performance results for all combinations of input variables, over all EEG channels are presented in Figure 4.

Statistical analysis of BCI performance based on classifier type (LDA vs. SVM) and number of SEP trials used for averaging showed that SVM classifier significantly outperformed LDA classifier [*F*(1,9) = 32.1, *p* = 0.0003], over all channels, sERP averaging methods, and feature extraction/selection methods. Also, statistically significant differences were obtained for comparison of sERP averaging options [SEP3 vs. SEP5 vs. SEP10, *F*(2,18) = 45.8, *p* = 0.0000008] with higher accuracies obtained for higher number of averaged trials (Figure 4), since single trial averaging increases sERP signal to noise ratio. The main effects of 2-way repeated measures ANOVA were significant but the interaction between factors was not significant [classifier type vs. number of trial for averaging, *F*(2,18) = 3.13, *p* = 0.068].

For the next analysis step, we narrowed our choice of methods to SVM classifier and SEP10 averaging, since those options resulted in better BCI performance (over 70%). Consequently, the combination of SVM-SEP10 and 4 different feature vector generation methods from 2 feature extraction approaches, with and without feature selection (F1, F2, FS1, and FS2), were tested with significant differences identified [*F*(1.63, 14.7) = 9.04, *p* = 0.004]. Results of the *post-hoc* comparison are given in Table 1. The results show significant increase in performance for both feature extraction approaches when feature selection is included.

**TABLE 1:** Results of the *post-hoc* comparison of BCI performance achieved with four feature extraction and selection methods for SVM classifier and SEP10 averaging option.

Comparison of BCI performance between methods	Test statistics	*P*-value
F1 vs. F2	1.22	0.252
FS1 vs. F2	4.42	**0.001**
F1 vs. FS1	−4.58	**0.0013**
F2 vs. FS2	−6.28	**0.0001**
F1 vs. FS2	−1.23	0.251
FS1 vs. FS2	2.07	0.06

Statistically significant differences are presented with *p*-value bolded.

Finally, the Table 2 summarizes the results for all subjects and EEG channels, for the optimal method, previously identified based on the best median performance: SVM classifier, SEP10 averaging, and feature extraction/selection–FS1. Table 3 shows classification accuracies separated for different target conditions presented by confusion matrices (for previously identified best channel of each subject).

**TABLE 2:** The BCI performance for all subjects and EEG channels obtained by the optimal feature extraction/selection and classification approach (SVM classifier, SEP10 averaging, and feature extraction/selection–FS1).

ID	Amp. D/V (mA)	C3	CP3	Cz	Pz	C4
1	17/12	80.9	**88.1**	84.6	65.3	63.8
2	13/12	**82.6**	81.4	73.4	72.0	72.2
3	17/12	80.9	75.0	**82.1**	78.5	78.6
4	11/11	**80.3**	73.3	77.9	73.3	67.5
5	10/7	71.6	71.6	**75.9**	71.6	60.1
6	11/8	77.9	73.3	**81.3**	77.9	80.2
7	12/12	64.8	**75.1**	52.2	75.0	50.1
8	11/10	70.9	73.3	**77.8**	70.8	67.8
9	17/12	80.4	82.3	80.3	**84.3**	80.3
10	17/17	**80.4**	74.2	71.7	71.8	74.0
Median {Q1-Q3}	12.5 {11–17} /12 {10–12}	80.35 {71.6–80.9}	74.6 {73.3–81.4}	77.85 {73.4–81.3}	72.65 {71.6–77.9}	70 {63.8–78.6}

Columns include subject ID, stimulation amplitude values at both D and V locations, and accuracies for 5 EEG channels. Highest accuracy per subject is presented in bolded number.

**TABLE 3:** Best BCI channel-performance per subject (SVM classifier, SEP10 averaging, and feature extraction/selection–FS1) and confusion matrices.

ID	Channel	Acc (%)	Confusion matrices (%)	
			**TP(D)**	**FP(D)**
			**FP(V)**	**TP(V)**
1	CP3	88.1	47.1	3.9
			8	41
2	C3	82.6	45.4	4.6
			12.8	37.2
3	Cz	82.1	42.5	8.2
			9.7	39.6
4	C3	80.3	41.2	8.8
			10.9	39.1
5	Cz	75.9	36.9	12.6
			11.5	39.1
6	Cz	81.3	40.7	9.2
			9.5	40.6
7	CP3	75.1	38.8	10.5
			14.4	36.3
8	Cz	77.8	37.6	11.8
			10.4	40.2
9	Pz	84.3	42.3	7.7
			8	42
10	C3	80.4	41	8.9
			10.7	39.4
Median {Q1–Q3}		80.85 {77.8–82.6}	41.1 {38.8–42.5}	8.85 {7.7–10.5}
			10.55 {9.5–11.5}	39.5 {39.1–40.6}

TP(D), percentage of correctly classified D targets; TP(V), percentage of correctly classified V targets; FP(D), percentage of D targets misclassified as V; FP(V), percentage of V targets misclassified as D.

## 4. Discussion

We developed and tested a novel electrotactile BCI prototype with control paradigm based on tactile attention task and sERP as BCI control-signal. Electrotactile BCI system comprises a commercial EEG amplifier, electrical stimulation device and a custom MATLAB based software for signal acquisition and device control. We tested a feasibility of single-channel EEG control, selected among 5 EEG channels. Our design also includes ES electrodes configuration comprising common indifferent stimulation electrode on the wrist and two spatially proximal locations of active stimulation electrodes positioned on the forearm, for stimulating the muscles innervated with mixed branches of radial and median nerves.

Our analysis comprised 3 sERP averaging approaches (3, 5 and 10 consecutive SEP trials), 2 feature extraction methods (F1, F2), effect of feature selection/fine-tuning, and 2 commonly used classifiers in BCI systems. Due to multiple factors, we have adopted a stepwise approach in our analysis. First step was to select a sERP averaging approach and classifier type. Analysis showed that SVM significantly outperformed LDA classifier and that only SEP10 averaging increased BCI performance over 70%. Moreover, our results showed statistically significant increase in classification accuracy with the increase of number of averaged SEPs (Figure 4). This result was expected since single-trial SEP averaging increases signal to noise ratio which is reflected in BCI performance. Moreover, this result further confirms the neurophysiological origin of the classified signals, since increase of accuracy with the increase of averaged single trials is expected in physiologically relevant data, contrary to the noisy signals. However, SEP5 and SEP3 approaches resulted in median classification accuracies below 70%, and therefore we used SEP10 averaging in the next analysis steps. Finally, the results show significant increase in performance for both feature extraction methods (F1 and F2) when feature selection is included (in combination with SVM classifier), and that FS1 approach achieved highest median accuracy.

Single-channel analysis summarized in Table 2 shows that BCI performance based on SVM, SEP10, and FS1 exceeded 75% in all subjects for at least one EEG channel while six subjects achieved accuracies over 80%. However, the EEG channel giving highest accuracy varied between subjects. On average, highest performance was achieved with C3, followed by Cz, CP3, Pz, and C4. The results show considerable variability among channel locations yielding the highest classification accuracy. Channel C3 with highest median accuracy was identified as the optimal in 3 subjects, CP3 in 2 subjects, Cz in 4 subjects, Pz in 1 subject and C4 was not selected.

Table 3 shows classification accuracies separated for different target conditions. The results are presented as confusion matrices including: percentages of correctly classified D targets, percentages of correctly classified V targets, percentages of D targets misclassified as V, and percentages of V targets misclassified as D. The results show that accuracies are balanced over conditions with the median value of correctly classified D targets is 41%, while the median value of correctly classified V targets is 40%, out of 50% stimuli delivered per each condition.

The results of this study are an important step toward an optimized online electrotactile BCI control where the cross-validation based approach is implemented for calibration of the device, selection of the optimal feature set, classifier training and control-channel selector. It is important to note that the achieved results were obtained in completely naive subjects without any training of the selective tactile attention task and without any feedback on their performance. Therefore, it might be expected that the performance could further increase with training of the subject and feedback inclusion during online BCI control. Regarding the prospects of online control, it is important to emphasize that results obtained *via* leave-one-out cross-validation are a realistic measure of online performance. The artifact rejection methods based on thresholds for EEG and EOG channels are easily applicable in an online scenario as well as the rest of the processing steps which were implemented in such manner to completely mimic the online BCI application.

Our BCI design comprises unique combination of electrotactile single pulse stimulation for eliciting SEPs for single-channel BCI control, spatially proximal locations of the stimulation sites on the same arm and stimuli probability balanced across 2 conditions. Consequently, the mismatch between conditions is created solely by the mental focus and not the difference is stimulus probability like in P300 studies employing oddball paradigm with lower probability of the target stimulus (Chu et al., 2021). This study also introduces a completely novel concept of sERP utilization for BCI control. In our approach the cortical representations of D and V locations in the contralateral somatosensory map are very close. Nevertheless, the sequential approach of single pulse stimulation allowed us to estimate the sERP responses associated with each of the stimuli sites, and consequently, generated within each of the neighboring cortical representations individually (even with the same EEG channel).

Previous reports on BCI control based on somatosensory electrical stimulation, used predominantly steady-state somatosensory evoked potentials (SSSEP) (Müller-Putz et al., 2006) while our electrotactile BCI platform is based on classification of SEPs averaged over consecutive single-trial responses for different stimuli locations, which is a completely novel approach enabling stimulation locations to be on the same limb unlike SSSEP-BCIs, which are based on detection of activation of more distant brain regions, exp. left vs. right hand sensorimotor areas (Petit et al., 2021). Moreover, SSSEP detection in all reviewed studies was based on multichannel EEG analysis, due to the fact that stimulation hotspots positioned on distant body parts elicit responses originating from different brain regions, requiring multiple EEG channels for recording the brain activity necessary for recognition (classification) of those signals [for review see Petit et al. (2021)].

Yao et al. (2013) explored the effects of vibrotactile stimulation in combination with selective sensation task as an alternative BCI task complementary to motor imagery. In these studies, event-related desynchronization/synchronization (ERD/ERS) in correlation with processing of afferent inflow in human somatosensory system, and attentional effect which modulated the ERD/ERS were explored.

The main methodological difference of our approach compared to previous studies using tactile stimulation and selective sensation approach is the introduction of stimulation locations at proximal locations of the same limb which wasn’t explored neither within SSSEP nor tactile ERD/ERS paradigms. Additionally, our approach is methodologically different from other tactile BCIs that utilize ERPs (mainly P300) for control, since we employed equal probability of target and distractor stimulus occurrence. This approach is different from classical oddball paradigm employed in other tactile ERP BCIs where the target (deviant, odd) stimuli is less frequent than the standard, and different from studies employing several targets of the same, smaller, probability compared to distractor stimuli (Guger et al., 2017; Spataro et al., 2018; Yao et al., 2022; Zhong et al., 2023). Guger et al. (2017) and Spataro et al. (2018) in one of their paradigms utilized vibrotactile stimulation on left/right wrists (targets) and distractor on the shoulder or back in order to induce P300 responses. The distractor received 75% of the stimuli, while the left and right wrist each received 12.5% of the stimuli. Their design involved equal probabilities of the targets, but the inclusion of the distractor with higher probability creates an oddball-like design to elicit P300.

Our sERP-based BCI introduces a compact design of 2 stimulation channels, requiring only 3 stimulation electrodes positioned on the same forearm for stimulating two mixed nerve branches (radial and median nerve). Intensities of ES for locations D and V varied between the subjects. The protocol included balancing of the pulse amplitudes between the locations in order to obtain similar subjective feeling of stimulation intensity. The amplitude for D site (10–17 mA) was higher or equal to the one used for V site (7–17 mA), as shown in Table 2. During the preliminary tests of the system, it was observed that the balancing of the stimulation intensity among the stimulation sites makes the tactile attention task easier since the attention is more easily kept on the stronger stimulus if the intensities are unbalanced.

Reports on use of steady-state somatosensory evoked potentials as BCI control signals claim that main advantage and reason for somatosensory stimuli introduction in BCI control is to overcome one of the greatest challenges of visual attention BCIs (that use P300 or steady-state visually evoked potentials) which is inherent visual fatigue after prolonged use (Ahn et al., 2016), and our work is in line with such recommendations. This BCI platform is adapted to requirements of both sensory training in restorative BCI (stroke or brain injury) applications and assistive BCIs, such as enabling communication in locked-in patients.

Information transfer rate (ITR) of our BCI design (number of targets: 2, number of commands: 1, time in seconds per decision for SEP3: 4.2 s, SEP5: 7 s, and SEP10: 14 s) is 14.29 bpm for SEP3, 8.57 bpm for SEP5, and 4.29 bpm for SEP10, respectively (Dal Seno et al., 2010). These results are comparable to ITR reported in very limited number of tactile ERP-based BCIs. Li et al. (2019) report ITR of 2.9 bpm in their P300 BCI based on somatosensory electrical stimulation for achieving the accuracy of 80% (4 EEG channel BCI design). Mao et al. (2021) reports ITR of 4.61–6.95 bpm (64.5–75.5% accuracy) of tactile P300 BCI using vibration stimuli (14 EEG channels). Chu et al. (2021) reports ITR of 6.75 bpm (94% accuracy) for vibration and 6.88 bpm (95% accuracy) for electrical stimuli in their P300 BCI design (14 EEG channels).

Our BCI design includes 5 EEG channels, however, the obtained accuracies are calculated for single-channel BCI control. Therefore, in an online scenario, a control channel can be selected among 5 EEG channel candidates with the best accuracy obtained during the classifier training. We hypothesize that the accuracy or ITR in our design can be further increased by multichannel control which may reduce the number of trials averaged to achieve higher accuracies. This will be a subject of future research. Moreover, the interstimulus interval of our BCI design is 700 ms, but the SEP pre-processing used for feature extraction is conducted on 500 ms epochs (100 ms baseline and 400 ms post-stimulus interval). Therefore, the shortening of ISI for increasing the ITR may be feasible and could be a topic for future research.

## 5. Conclusion

We presented a feasibility of a novel electrotactile BCI platform. Our BCI design is compact including novel stimulation setup consisted of 2 electrical stimulation channels positioned on a forearm of the same limb. Our control paradigm is based on selective tactile attention toward a chosen target location, with equal probability of stimulus occurrence. The control signals driving the system are SEPs elicited by a single electrical pulse stimulating mixed radial or median nerve branches which enables the proximity of the stimulation locations positioned on the same limb unlike SSSEP BCI control. We presented a dedicated feature extraction and selection methods and results showing that single EEG channel BCI performance offline estimation in all subjects ranged from 75.1 to 88%. We propose this method for driving restorative BCIs for sensory retraining in stroke or brain injury or assistive BCIs for communication in severely disabled users.

## Data availability statement

The data that support the findings of this study will be made available by the corresponding author AS, andrej_savic@etf.rs, upon reasonable request.

## Ethics statement

The studies involving human participants were reviewed and approved by Ethics Committee of the Clinic for Rehabilitation “Dr. Miroslav Zotović”. The participants provided their written informed consent to participate in this study.

## Author contributions

AS conceived the BCI control concept, wrote the manuscript, and managed the project/study. MN and AS performed the experiments, collected the data, and developed the software for signal acquisition and hardware control. VM-J and AS performed the offline data processing and analysis. AS, OĐ, and LK contributed to the experimental protocol design and ethical approval obtainment. All authors reviewed the final manuscript.
